# Long-Term Survival Rate of ATOMS Implant for Male Stress Urinary Incontinence and Management of Late Complications

**DOI:** 10.3390/jcm12062296

**Published:** 2023-03-15

**Authors:** Alessandro Giammò, Enrico Ammirati

**Affiliations:** CTO-Spinal Cord Unit, NeuroUrology Department, Città della Salute e della Scienza di Torino, 10126 Turin, Italy

**Keywords:** male stress urinary incontinence, ATOMS system, post-prostatectomy urinary incontinence, long-term results

## Abstract

Background: stress urinary incontinence (SUI) still represents a major drawback of prostate surgery. The aim of this study is to evaluate long term efficacy, safety and survival of ATOMS system implant in a single center. Methods: we retrospectively included al consecutive patients treated with ATOMS implant for SUI from October 2014 to July 2019. Patients received anamnesis, urodynamic evaluation, pre- and postoperative 24 h pad test and count. Patients were considered “continent” when dry or when wearing a security pad (social continence). Results: we treated99 patientswith median age 77.98 years (IQR 72.7–82.52). Most of the patients had undergone radical prostatectomy. Median follow-up was 62.9 months (IQR 47.5–75.9). At last follow-up 74 (74.7%) patients reported continence. We had 21 early (<30 days) postoperative complications, all Clavien-Dindo (CD) grade 1 [11 temporary perineal pain, 4 urinary retention, 3 scrotal edema, 2 superficial wound dehiscence, 1 dysuria]. We had late postoperative complications in 28 patients [7 port dislocations requiring surgical repositioning (CD 3a), 6 device removals (CD 3a) due to port erosion (2), inefficacy (2), cushion leakage (1), mesh detachment (1), perineal pain (5), 2 cases of port extrusion solved with port removal leaving the device in place (CD 3a), 2 superficial wound dehiscence (CD 1), 2 UTI (CD 1), 1 scrotal edema (CD 1), 1 cushion deflate (CD 1), 1 dysuria (CD 1), 1 perineal pain (CD 1)]. The survival of the device was 97% at 12 months, 93% at 24 months, 91% at 36 months, 90% at 48 months and 87.9% at 60 months. Conclusions: This study demonstrates the good safety and efficacy of ATOMS implant for the treatment of SUI.

## 1. Introduction

Post-prostatectomy stress urinary incontinence (SUI) still represents a rather frequent complication. In an extensive review of the SEER cancer registry, Kim et al. collected data on 16,348 men older than 66 years who had undergone radical prostatectomy surgery; 1057 (6%) patients in this population underwent at least one surgery for urinary incontinence [1].

The artificial urethral sphincter (AUS) is widely considered the reference standard technique for the treatment of male SUI. This device has demonstrated to have an efficacy in term of continence up to 86%. The high costs of the device, the incidence of postoperative complications and the relatively high revision rates have led to the development of male slings and compressive devices [2]. Researchers have developed fixed and adjustable suburethral transobturator slings and adjustable compressive devices. Adjustable devices may give the possibility of post-implant tensioning, augmenting the urethral compression when SUI persist after device positioning.

The ATOMS system is an adjustable sling-shaped transobturator compression device that determines a non-circumferential urethral compression and the possibility of postoperative adjustments. The device consists of a silicone cushion which is placed over the bulbourethral muscle, compressing the urethra, anchored to the pubo-ischiatic branches with two arms in a macroporous monofilament polypropylene mesh. A dedicated tunneler allows an out–in transobturator passage. The cushion is connected to a titanium port that is placed in the scrotum, allowing for percutaneous postoperative adjustments with a saline injection until continence is achieved in an out-patient setting.

The studies to date show results in terms of the effectiveness and safety of the device only with a short- and medium-term follow-up. The aim of this study is a long-term evaluation (follow-up > 36 months) of the safety and survival of the device.

## 2. Materials and Methods

This study includes all consecutive patients with SUI who underwent an ATOMS implant from October 2014 to July 2019 in a single high-volume center. All interventions were performed by a single surgeon. All patients had received preoperative counseling, including the evaluation of an AUS implant; all degrees of SU were offered ATOMS and AUS, with the decision made according to surgeons’ and patients’ preferences. All patients were implanted with third-generation devices with a scrotal port. The following data were retrospectively collected for each patient on their clinical records: medical history, physical examination, preoperative urodynamic investigation, preoperative and post-operative 24 h pad test and pad count, and voiding diary. For each patient, a first postoperative follow-up control was scheduled after one month for pressurization of the device in case incontinence persisted; subsequent follow-up controls and any subsequent pressurization of the device did not follow a fixed protocol, but were adapted to the patient’s needs.

The primary outcome of this study is the evaluation of the safety of the device and its explantation-free survival. The secondary outcome is the evaluation of the effectiveness of the device, based on the reduction of the 24 h pad test and the pad count. We defined a patient as being continent when dry or using a single daily pad (social continence).

Quantitative variables were expressed as medians and interquartile range (IQR), while qualitative variables were expressed as numbers and percentages. The 24 h pad test and pad count data are not normally distributed (Kolmogorov–Smirnov normality test < 0.001); thus, nonparametric tests were applied. A sub-group analysis was performed for patients with an RT history, previous urethral surgery, and previous incontinence surgery. We applied a linear mixed-effect model (residual maximum likelihood REML, t-tests, Satterthwaite’s method). Statistical significance was established with a p value < 0.05.

## 3. Results

We included 99 patients in this study. The median age was 77.98 years (IQR 72.7–82.52 years). Most patients underwent surgery for prostate cancer; in one case, a patient had undergone only radiotherapy (RT) for prostate cancer, and in another, a patient was affected by cauda equina syndrome. Half of the population examined had undergone previous surgery for urinary incontinence. Table 1 summarizes the preoperative data.

The median follow-up was 62.9 months (IQR 47.5–75.9 months). Early postoperative complications (within the first 30 days of implantation) occurred in 21 (21.2%) patients. All early complications were low-grade (Clavien–Dindo 1). Late postoperative complications (>30 days after implantation) occurred for 28 (28.3%) patients (Table 2).

**Figure 1:**
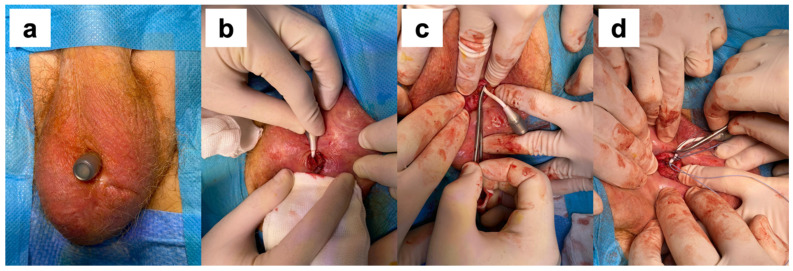
(**a**) Extrusion of the port; (**b**) exposure of the connection tubing to the port; (**c**) clamping of the connection tubing; (**d**) placement of non-absorbable sutures before proceeding with the section and removal of the port.

**Figure 2:**
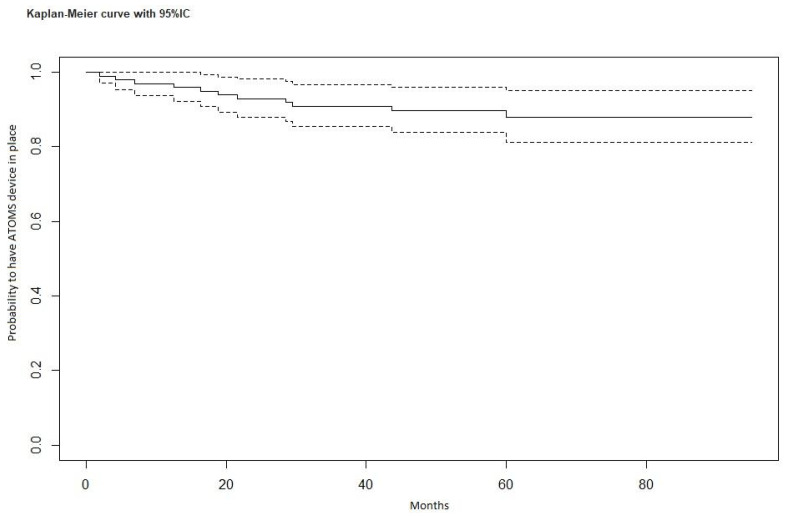
Survival of the device, Kaplan–Meier curve with 95% confidence interval.

A surgical procedure was required in 20 cases, including 7 cases of port revision (keeping the device in place), 2 cases of extruded port removal (keeping the device in place) (Figure 1), and 11 cases of complete device removal. The breakage of the silicone cushion and the detachment of the polypropylene arms were intraoperative findings during the surgical removal of the device in patients with failure to improve continence. Port revision was performed on patients with dislocation of the port that precluded further cushion filling. After a surgical incision, the port was isolated and repositioned in a sovra-dartoic scrotal position. We considered port removal for early extrusion for device survival because the cushion was still in place and the device was still exerting its compressive action. The median time between an ATOMS device implantation and re-operation was 16.5 months (IQR 7.1–29.4 months). The probability of having the device in place at 12 months is 97%; at 24 months, 93%; at 36 months, 91%; at 48 months, 90%; and at 60 months, 87.9% (Figure 2).

The median number of cushion refills was 2 (IQR 1–4), with a median cushion filling volume of 12 mL (IQR 6–19 mL). At the last follow-up, we found a significant reduction in the 24 h pad test (*p* < 0.00001) and daily pad count (*p* < 0.00001). A total of 74 (74.7%) patients reported continence. Continence results regarding the global population and in patients with a history of RT and previous urethra and incontinence surgery are included in Table 3. Preoperatively, there was no significant difference in terms of pad testing between subgroups.

Applying the linear mixed-effect model (residual maximum likelihood REML, *t*-tests, Satterthwaite’s Method), we did not find a statistically significant change in the 24 h pad test or pad count in patients undergoing RT (*p* = 0.44 and *p* = 0.55) or previous urethral surgery (*p* = 0.68 and *p* = 0.88) when compared to patients who did not undergo RT or previous urethral surgery, respectively; instead, we found worse continence results in terms of the 24 h pad test (+50 g, *p* = 0.035) and pad count (+0.54, *p* = 0.005) in patients who had had previous continence surgery, compared to patients without previous incontinence surgery.

## 4. Discussion

This study comprehends a population of 99 patients affected by SUI and treated with the ATOMS device implant. The median follow-up was 62.9 months. The device showed good survival, with a 60-month patient implant rate of 87.9%. The device was demonstrated to be safe, with an early complication rate (within the first 30 days of implantation) of 21.2% and a late postoperative complication rate (more than 30 days of implantation) of 28.3%. All early complications were low grade (Clavien–Dindo 1). Regarding late complications, in 20/28 cases, a surgical procedure was required, which included 7 cases of port revision (keeping the device in place), 2 cases of extruded port removal (keeping the device in place), and 11 cases of complete removal of the device. To reduce the risk of scrotal port displacement, we modified the implant technique, applying a closing suture to close the digitally created scrotal channel. It is possible that this change in the implant technique will lower the need for port revision in the future. We report that five cases of device removal occurred due to discomfort from the device by patients who complained of a sensation of tension or pain in the perineal area. The complication rate appears in line with the data available in the literature (16.4–33.7%) [3,4,5].

We adopted a conservative treatment in two cases of scrotal port erosion, removing the port and keeping the cushion in place. According to good clinical practice, it is imperative to remove the entire device in case of extrusion due to the high risk of infection. For instance, two patients opposed the removal of the device, having achieved an excellent state of continence, and accepted the procedure after appropriate counseling. In both cases, the port was removed under local anesthesia, with the residual portion of the connections closed with non-absorbable sutures and full-dose antibiotic therapy with cephalosporins continued for a week (Figure 2). In both cases, there were no complications after the procedure, there were no episodes of device infection, and the previously achieved state of continence was maintained.

This study highlights the long-term effectiveness of the ATOMS device with a continence rate of 74.7%. Patients who underwent previous urethral surgery and RT did not show statistically significant changes in continence. Previous incontinence surgery, on the other hand, showed inferior results; therefore, a careful choice of the first device is mandatory to optimize continence results. The effectiveness is comparable to that given in a systematic literature review by Esquinas et al. on 20 different studies (13 retrospective and 7 prospective, of which 3 are multicenter) with 1393 patients. The authors reported a 67% (95% CI 0.61–0.72) mean dry rate and a 90% (95% CI 0.86–0.94) mean improvement rate, with a significant reduction in the pad count (mean −4.14, 95% CI −4.52/−3.76) and 24 h pad test (mean −443 g, 95% CI −482.6/−403.5 g) [5].

To give an overview on the efficacy and safety of the other surgical options for male SUI treatment, we are reporting the results of a systematic review including 13,100 patients. A total of 3 papers reported the results of bulking agents; 35 were on synthetic male slings, 10 on ProACT (adjustable continence therapy) devices, and 37 on AUS. Sling surgery was correlated to a reoperation rate of 5.8% (95% CI: 1.9–11.6, I2 = 94.1%, moderate level of evidence), ProACT 23.8% (95% CI: 5.9–61.0, I2 = 95.5%, level of evidence), AUS 22.2% (95% CI: 15.2–31.3, I2 = 92.3%, high evidence). The efficacy of bulking agents was 26.1% (95% CI: 10.6–51.4, I2 = 92.8%, a very low level of evidence), slings 58.6% (95% CI: 51.3–65.5, I2 = 89.1%, a low level of evidence), ProACT63.2% (95% CI: 57.6–68.5, I2 = 22.5%, a very low level of evidence), and AUS 74.0% (95% CI: 61.2–83.7, I2 = 92.1%, a very low level of evidence) [6].

Two different studies compared the effectiveness and safety of the ATOMS system with ProACT and Reemex devices. ATOMS seemed to have a higher efficacy than ProACT (dry rate 68%, 95% CI 62–73 vs. 55%, 95% CI 47–63, *p* = 0.01; improvement rate 91%, 95% CI 87–94 vs. 80%, 95% CI 72–87, *p* = 0.007) and Reemex devices (dry rates 69.3%, 95% CI 63.9–74.4 vs. 53.4%, 95% CI% 42.2–64.3, *p* = 0.008, improvement rates 90.8%, 95% CI 86.6–94.4 vs. 80.2%, 95% CI 71.5–87.8, *p* = 0.007). The explant rate was lower for the ATOMS system than for the ProACT (5%, 95% CI 2–9 vs. 25%, 95% CI 19–31, *p* < 0.0001) and Remmex devices (5.4%, 95% CI 2.8–8.8 vs. 13.9%, 95% CI 6.6–22.9, *p* = 0.027) [7,8].

The strength of this study is the size of the population, the length of the follow-up, and the lack of missed patients at follow-up; we had pad test data for all patients at the last follow-up. The main limitations are the retrospective nature of the study and the lack of standardized questionnaires for all patients; there is a possible selection bias as all patients were offered the opportunity to choose between ATOMS and AUS implants.

## 5. Conclusions

This study demonstrates the safety and effectiveness of the ATOMS device for the treatment of male stress urinary incontinence in the long term. With a median follow-up of 62.9 months, the results appear in line with the literature data. We reported a late complication rate of 28.3% and a surgical revision rate of 20.2%. At 60 months, it is estimated that 87.9% of the devices are still in place, demonstrating good longevity. We reported a 74.7% continence rate at the last follow-up. Radiotherapy and previous urethral surgery do not seem to reduce continence results, whereas a previous incontinence surgery may slightly lower the efficacy of the device.

## Figures and Tables

**Table 1:** Baseline characteristics.

Previous Prostate Surgery (n)	
RRP	75
LARP	10
RARP	3
TURP	4
Simple prostatectomy	3
HoLEP	1
ThuLEP	1
Urethral surgery after prostate intervention (n)	
Urethrotomy	16
Bladder neck incision	6
Laser anastomotic incision	3
Adjuvant RT	16
Urodynamic data(median)	
Qmax	20 mL/sec (IQR 12.75–24.5 mL/sec)
pDetQmax	38 cmH_2_O (IQR 29.25–52.75 cmH_2_O)
Median bladder capacity	300 mL (IQR 300–400 mL)
Median VLPP	65 cmH_2_O (IQR 41.25–90cmH_2_O)
Previous incontinence treatments (n)	
Pelvic floor rehabilitation	29
Oral treatment	
- Duloxetine	5
- Antimuscarinics	7
- Both	8
Incontinence surgery:	
- ProACT	44
- AUS	2
- ProACT + AUS	3
- ProACT + bulking agent	1
- Sling	1

RRP, radical retropubic prostatectomy; LARP, laparoscopic-assisted radical prostatectomy; RARP: roboitc-assisted radical prostatectomy; TURP, transurethral resection of the prostate; HoLEP, holmium laser enucleation of the prostate; ThuLEP, thulium laser enucleation of the prostate; RT, radiotherapy; Qmax, maximum flow; pDetQmax; detrusor pressure at maximum flow; VLPP, Valsalva leak point pressure; IQR, interquartile range; ProACT, adjustable continence therapy; AUS, artificial urethral sphincter.

**Table 2:** Postoperative complications.

Early Postoperative Complications (n, %)	
Temporary perineal pain and paresthesia (Clavien–Dindo 1)	11 (11.1%)
Acute urinary retention that resolved with device deflate (Clavien–Dindo 1)	4 (4%)
Temporary scrotal edema (Clavien–Dindo 1)	3 (3%)
Superficial wound dehiscence not associated with infection (Clavien–Dindo 1)	2 (2%)
Dysuria resolved with device deflate (Clavien–Dindo 1)	1 (1%)
Late postoperative complications (n, %)	
Port dislocation requiring surgical revision (Clavien–Dindo 3a)	7 (7%)
Removal of the device(Clavien–Dindo 3a) due to:	11 (11.1%)
- Pain	5 (5%)
- Erosion of the port	2 (2%)
- Ineffectiveness	2 (2%)
- Breakage of the silicone cushion	1 (1%)
- Detachment of the polypropylene arms	1 (1%)
Port removal for early extrusion(Clavien–Dindo 3a)	2 (2%)
Wound dehiscence without device infection (Clavien–Dindo 1)	2 (2%)
Urinary tract infection (Clavien–Dindo 1)	2 (2%)
Scrotal edema (Clavien–Dindo 1)	1 (1%)
Cushion spontaneously deflated (Clavien–Dindo 1)	1 (1%)
Dysuria resolved with device deflation (Clavien–Dindo 1)	1 (1%)
Perineal pain and paresthesia (Clavien–Dindo 1)	1 (1%)

**Table 3:** Continence results.

Global Population	Preoperative	Postoperative
24 h pad test	350 g (IQR 300–400 g)	60 g (IQR 0–100 g)
Pad count	4 pads/day (IQR 3–5)	1 (IQR 0–1)
Continent patients	-	74 (74.7%)
Radiotherapy-treated patients (n = 16)		
24 h pad test	335 g (IQR 290–400 g)	50 g (IQR 27.5–87.5 g)
Pad count	3 pads/day (IQR 3–4)	1 (IQR 1–1)
Previous urethral surgery (n = 27)		
24 h pad test	350 g (IQR 300–400 g)	77.5 g (IQR 35–100 g)
Pad count	4 pads/day (IQR 3–5)	1 (IQR 1–1)
Previous incontinence surgery (n = 50)		
24 h pad test	380 g (IQR 305–437.5 g)	80 g (IQR 30–100 g)
Pad count	4 pads/day (IQR 3–5)	1 (IQR 1–2)
Naive population (n = 25)		
24 h pad test	350 g (IQR 200–400 g)	10 g (IQR 0–60 g)
Pad count	4 pads/day (IQR 2–5)	0 (IQR 0–1)
	Dry	Social continence
Global population	29/99 (29.3%)	45/99 (45.5%)
Radiotherapy treated patients	3/16 (18.7%)	10/16 (62.5%)
Previous urethral surgery	5/27 (18.5%)	16/27 (56.2%)
Previous incontinence surgery	10/50 (20%)	25/50 (50%)
Naive population	14/25 (56%)	6/25 (24%)

Naive population: no RT + no urethral surgery + no incontinence surgery.

## Data Availability

Data is unavailable due to privacy restrictions.
